# Evaluation of Models Describing the Growth of Nalidixic Acid-Resistant *E. coli* O157:H7 in Blanched Spinach and Iceberg Lettuce as a Function of Temperature

**DOI:** 10.3390/ijerph10072857

**Published:** 2013-07-09

**Authors:** Juhui Kim, Hyunjung Chung, Joonil Cho, Kisun Yoon

**Affiliations:** 1 Department of Food and Nutrition, Kyung Hee University, 1 Hoeki-dong, Dongdaemun-gu, Seoul 130-701, Korea; E-Mail: wngml2316@gmail.com; 2 Department of Food and Nutrition, In Ha University, 253 younghyun-dong, Nam-Gu, Incheon 402-751, Korea; E-Mail: hjchung@inha.ac.kr; 3 Food Microbiology Division, National Institute of Food and Drug Safety Evaluation, Ministry of Food and Drug Safety, Osong-eup 363-700, Korea; E-Mail: kvoyou@korea.kr

**Keywords:** nalidixic acid-resistant *E. coli* O157:H7, blanched spinach, fresh cut iceberg lettuce, growth model

## Abstract

The aim of this study was to model the growth of nalidixic acid-resistant *E. coli* O157:H7 (*E. coli* O157:H7^NR^) in blanched spinach and to evaluate model performance with an independent set of data for interpolation (8.5, 13, 15 and 27 °C) and for extrapolation (broth and fresh-cut iceberg lettuce) using the ratio method and the acceptable prediction zone method. The lag time (LT), specific growth rate (SGR) and maximum population density (MPD) obtained from each primary model were modeled as a function of temperature (7, 10, 17, 24, 30, and 36 °C) using Davey, square root, and polynomial models, respectively. At 7 °C, the populations of *E. coli* O157:H7^NR^ increased in tryptic soy broth with nalidixic acid (TSBN), blanched spinach and fresh-cut iceberg lettuce, while the populations of *E. coli* O157:H7 decreased in TSB after 118 h of LT, indicating the risk of nalidixic acid-resistant strain of *E. coli* O157:H7 contaminated in ready-to-eat produce at refrigerated temperature. When the LT and SGR models of blanched spinach was extended to iceberg lettuce, all relative errors (percentage of RE = 100%) were inside the acceptable prediction zone and had an acceptable *B_f_* and *A_f_* values. Thus, it was concluded that developed secondary models for *E. coli* O157:H7^NR^ in blanched spinach were suitable for use in making predictions for fresh cut iceberg lettuce, but not for static TSBN in this work.

## 1. Introduction

Fresh vegetables, such as radish sprouts, pre-packed spinach, and lettuce have been recognized as a major vehicle associated with outbreaks of illness caused by *Escherichia coli* O157:H7 [1,2]. In a USDA Agricultural Marketing Service survey, 29 spinach samples out of 2,328 fresh produce items available at terminal markets and wholesale distribution centers were identified as positive for pathogenic *E. coli* by mPCR [1]. A multistate outbreak of *E. coli* O157:H7 infections linked to Romaine lettuce was also reported in the USA [3]. The virulent strain of *E. coli* O104:H4 in beansprouts has killed 35 and sickened 3,256 in Europe [4]. A total of 33 ill persons infected with an outbreak of the strain *E. coli* O157:H7 which was linked to pre-packed leafy greens were also reported [5]. According to a report on foodborne outbreaks in Canada [6], the food vehicles most commonly implicated in outbreaks were leaf greens and herbs (26%), followed by seed sprouts (11%).

Predictive models that describe the behavior of *E. coli* O157:H7 have been developed in broth based on temperature, pH, and additives, such as salt and sodium nitrite [7,8,9,10,11,12]. As fresh produce has been identified as a vehicle for foodborne illness due to *E. coli* O157:H7 contamination, a complete predictive model for the growth of *E. coli* O157:H7 has only been developed for iceberg lettuce at constant temperatures ranging from 5 to 25 °C [13]. Recently, a combined growth-death model for *E. coli* O157:H7 in minimally processed leafy green vegetables using the data from 13 studies was developed [14]. The result showed that the Koseki and Isobe [13] data points, such as growth rate, were the highest of any of the published data sets at all temperatures with under prediction at low temperature, and over prediction at high temperature. Thus more predictive models for *E. coli* O157:H7 for various forms of produce, including fresh and processed produce, could be a valuable tool to estimate the microbial risk of such foods during distribution.

An important step in the development of predictive models is an evaluation of model performance, which involves goodness-of-fit, interpolation, and extrapolation (model extension). Data on the performance evaluation of interpolation are collected with the same strain, growth media, and modeling methods but by using different combinations of the independent variables that are within the response surface of the model. Also, data for performance evaluation of extrapolation (model extension) are collected in the same manner except that the growth medium or other strains are varied [15]. In general, the performance of a model is quantified using the ratio method [16] and the acceptable prediction zone method [17].

In the present study, we investigated and modeled the growth kinetics of nalidixic acid-resistant *E. coli* O157:H7 (*E. coli* O157:H7^NR^) in broth, blanched spinach, and fresh-cut iceberg lettuce. Additionally, the model performance of the blanched spinach model was evaluated with data not used during model development: an independent set of data for interpolation (at 8.5, 13, 15 and 27 °C) and for extrapolation (using broth and fresh-cut iceberg lettuce model) using the ratio method and the acceptable prediction zone method.

## 2. Experimental Section

### 2.1. Bacterial Strains

The strains of *E. coli* O157:H7 (NCTC12079 and ATCC35150) were maintained at −80 °C in tryptic soy broth (TSB, DIFCO, Sparks, MD, USA) that contained 20% glycerol (Sigma-Aldrich, St. Louis, MO, USA). In this work, we used a nalidixic acid-resistant *E. coli* O157:H7 strain to distinguish inocula from background microorganisms, including *E. coli.* The nalidixic acid-resistant strains were prepared according to the method of Inatsu *et al.* [18] and were designated as *E. coli* O157:H7^NR^. *E. coli* O157:H7 was grown in TSB supplemented with nalidixic acid (Sigma Chemical, St. Louis, MO, USA, 50 µg/mL TSBN). Cultures were incubated at 36 °C and transferred (1 loopful to 10 mL TSBN) three times at 24 h intervals and stored at −80 °C in TSBN containing 20% glycerol. Ten microliters of thawed stock culture was inoculated into a 50 mL Erlenmeyer flask containing 10 mL of TSBN, which was then sealed with a silicone cap and incubated at 36 °C for 24 h on rotary shaker (VS-8480SR, Vision, Daejeon, Korea) at 140 rpm. Viable cell counts ranged from 8.5–9.5 log(CFU/mL).

### 2.2. Preparation of Inocula for the Broth Model

A volume of TSBN (25 mL) was prepared in a 250 mL Erlenmeyer flask. Each TSBN sample was aseptically inoculated with the diluted starter of a mixture of two *E. coli* O157:H7^NR^ strains (NCTC12079^NR^ and ATCC35150^NR^) to give a target population of approximately 2.5–3.5 log(CFU/mL). Inoculated broths were then stored without agitation, which was comparable to the growth dynamics of the pathogen in a more static food medium [19].

To quantify growth during the storage period, diluted culture (100 µL) was plated onto tryptic soy agar (TSA, DIFCO) supplemented with 50 µg nalidixic acid/mL TSAN in duplicate, respectively, and incubated aerobically at 36 °C for 24 h. The colonies on TSAN plates were then counted and bacterial counts from duplicate plates were converted to log numbers.

### 2.3. Preparation and Inoculation of Blanched Spinach and Fresh-Cut Iceberg Lettuce

Bundled spinach (*Spinacia oleracea L.*) and wrapped iceberg lettuce (*Lactuca sativa L.*) stored at 10 °C were purchased from a local market in Seoul, Korea and transported to the lab within 1 h. The stems and roots of bundled spinach were removed and only spinach leaves were washed with running tap water and blanched in 100 °C water for 1 min. Blanched spinach was divided into individual 10 g portions and aseptically placed in Petri dishes. The surfaces of blanched spinach leaves were then inoculated uniformly with 100 µL of the diluted starter culture of a cocktail mixture of *E. coli* O157:H7^NR^ strains using a sterile pipette to give a target population of approximately 2.5–3.5 log(CFU/g). The inoculated samples were then stored at 7, 10, 17, 24, 30 and 36 °C.

For inoculation of iceberg lettuce, the external, damaged iceberg lettuce leaves and core were discarded before washing. Lettuce leaves were individually washed twice with running tap water for 1 min then rinsed with sterilized distilled water for 1 min and cut into 10 g samples with a disinfected knife.

The fresh-cut lettuce leaves were immersed in inoculum suspension (2 L) for 3 min, which was prepared by transferring l mL of a cocktail mixture of *E. coli* O157:H7^NR^ strains into 2 L of sterile distilled water. After drying in a bio-safety cabinet for 1 h, 10 g of inoculated sample was aseptically divided into sterile bags and stored at 7, 10, 17, 24, 30 and 36 °C. Each sample was homogenized (Stomacher, Interscience, Paris, France) for 2 min in 40 mL of 0.1% sterilized peptone water. One milliliter of homogenized sample was diluted in 9 mL of 0.1% sterilized peptone water. One hundred microliter aliquots of two dilutions of each sample were plated on TSAN in duplicate and incubated aerobically at 36 °C for 24 h. The colonies on duplicate plates of each sample were counted, and then converted to log numbers.

### 2.4. Experimental Designs

Blanched spinach, fresh-cut iceberg lettuce, and tryptic soy broth supplemented with 50 µg nalidixic acid (TSBN) were used for model development as a function of temperature. The performance of the models for lag time (LT), specific growth rate (SGR), and maximum population density (MPD) of *E. coli* O157:H7^NR^ strains (a mixture of NCTC12079^NR^ and ATCC35150^NR^) in blanched spinach and fresh-cut iceberg lettuce were confirmed with data obtained at temperatures not included in model development.

Additionally, models with different growth substrates including fresh-cut iceberg lettuce and TSBN were compared to the growth model developed with a mixture of *E. coli* O157:H7^NR^ in blanched spinach for evaluation of applicability of the blanched spinach model for iceberg lettuce and TSBN (model extension).

### 2.5. Primary Modeling

Viable counts (log(CFU/g)) of *E. coli* O157:H7^NR^ were graphed as a function of time and then iteratively fitted to the modified Gompertz model using GraphPad PRISM V4.0 (GraphPad Software, San Diego, CA, USA) to determine the lag time (LT), specific growth rate (SGR), and maximum population density (MPD). The model used was as follows:

Y_t_ = N_0_ + C × exp{−exp[(2.718 × SGR/C) × (LT − t) + 1]} (1)

where Y_t_ is the viable cell count (log(CFU/g)) at time t(h), N_0_ is the initial log number of cells, C is the difference between the initial and final log cell numbers, SGR is the maximum specific growth rate (log(CFU/h)), LT is the lag time before growth, and t is the sampling time. The goodness-of-fit of the data was also evaluated based on the coefficient of determination (R^2^), which was provided by GraphPad Prism. 

### 2.6. Secondary Modeling

LT, SGR, MPD values were graphed as a function of temperature and then fitted to the Davey, square root, and polynomial models, respectively. The Davey model used was as follows [20,21]:

Y = a + (b/T) + (c/T^2^) (2)

where Y is LT(day), a, b, and c are regression coefficients without biological meaning, and T is temperature.

The square-root model used was as follows [22]:

√Y = b(T − Tmin)(3)

where Y is SGR (log(CFU/day)), b is a regression coefficient, T is temperature, Tmin is the cardinal minimum growth temperature.

The second order polynomial model used was as follows [23]:

Y = a + bT + cT^2^ (4)

where Y is MPD (log(CFU)), a, b, and c are regression coefficients without biological meaning, and T is temperature.

### 2.7. Performance Evaluation of Blanched Spinach and Fresh-Cut Iceberg Lettuce Models

The performance of the models was quantified using the ratio method described by Ross [16] and an acceptable prediction zone method [17]. Prediction bias (*B_f_*) and accuracy (*A_f_*) factors were calculated using the following equations [24].

*B_f_* for LT = 10^∑l^^og(predicted/^^observed)/n^ (5)

*A_f_* for LT = 10^∑|l^^og(predicted/^^observed)|/n^ (6)

*B_f_* for SGR = 10^∑l^^og(^^observed/predicted)/n^ (7)

*A_f_* for SGR = 10^∑^^|l^^og(^^o^^bserved/^^predicted)^^|/n^ (8)

where n is the number of prediction cases used in the calculation. Different ratios were used for LT and SGR, so that *B_f_* less than 1 would represent fail-safe predictions. In addition, relative errors (RE) of individual prediction cases were calculated using the following equation [25]:

RE for LT (%) = [(predicted – observed)/predicted] × 100(9)

RE for SGR (%) = [(observed – predicted)/predicted] × 100(10)

The median relative error (MRE) and the mean absolute relative error (MARE) were also used to measure the prediction bias and accuracy of the model, respectively. In the acceptable prediction zone method, the percentage of RE (%RE) that is in an acceptable prediction zone (*i.e.*, the ratio of the number of RE in the acceptable prediction zone to the total number of prediction cases) from −30 to 15% for SGR and −60 to 30% for LT, and −80 to 40% for MPD were calculated and used as a measure of model performance [15].

## 3. Results and Discussion

### 3.1. Development of a Predictive Growth Model for *E. coli* O157:H7^NR^ in Blanched Spinach and Fresh-Cut Iceberg Lettuce as a Function of Temperature

**Figure 1:**
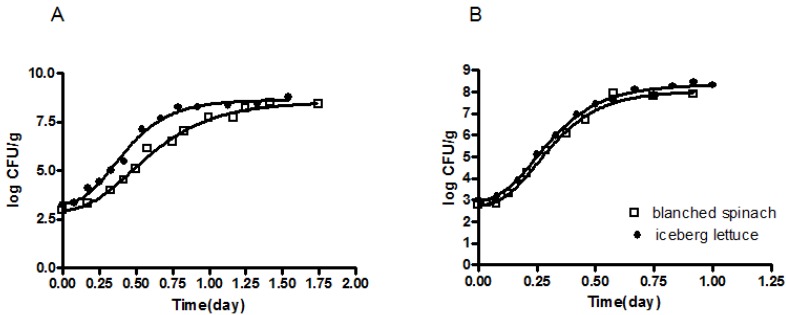
Representative primary growth model for *E. coli* O157:H7^NR^ in blanched spinach and fresh cut iceberg lettuce stored at (**A**) 24 and (**B**) 30°C.

A modified Gompertz equation was used as the primary growth model for *E. coli* O157:H7^NR^ in blanched spinach and fresh-cut iceberg lettuce, which were stored at 7, 10, 17, 24, 30 and 36 °C. Primary growth models for blanched spinach and fresh-cut iceberg lettuce were fitted well to a modified Gompertz equation with a high degree of goodness-of-fit (R^2^ = 0.9845 to 0.9988) (Figure 1). 

The effect of temperature on the growth parameters derived from the primary model for blanched spinach and fresh-cut iceberg lettuce was compared (Table 1). The LT of a mixture of *E. coli* O157:H7^NR^ increased as the temperature decreased. The LT of a mixture of *E. coli* O157:H7^NR^ on blanched spinach stored at 7 °C was approximately 26 times longer than that stored at 36 °C. The SGR of a mixture of *E. coli* O157:H7^NR^ at 10 °C and 24 °C was 35.2 and 2.2 times slower, respectively, compared to the SGR at 36 °C. In this study, LT and SGR of a mixture of *E. coli* O157:H7^NR^ in iceberg lettuce was 40.08 h and 0.051 log(CFU/h), respectively, at 10 °C, which was lower than the LT (32.64 h) and was higher than SGR (0.03 log(CFU/h)) in iceberg lettuce model developed by [13]. Bharathi *et al.* [26] reported the growth data of an enterotoxigenic strain of *E. coli* D21 in minimally processed vegetables (carrot, cucumber and tomato) at a variety of initial inocula (4.3, 5.3 and 6.3 log(CFU/g)), storage temperatures (2, 9 and 16 °C) and storage times (2, 6 and 10 days). In their study, an increase in the viable population of *E. coli* was observed at only 9 and 16 °C. Recently, Khalil and Frank [27] reported that *E. coli* O157:H7 population was greater on spinach when compared with other leafy greens (parsley, cilantro) at 8 °C after 3 days of storage. In addition, *E. coli* O157:H7 did not grow on damaged Romaine leaves at 8 or 12 °C, but growth was observed at 15 °C. These results confirm that the minimum growth temperature and growth rate varies with the strain of inoculated organisms and quality condition of the vegetables. Palumbo *et al.* [28] also studied the minimum growth temperatures for 16 strains of *E. coli* O157:H7 in BHI broth using a temperature gradient incubator. The minimum growth temperatures varied for the 16 strains: 11.6 °C (two strains), 11.3 °C (one stain), 9.4 °C (five strains), 9.2 °C (seven strains), and 7.3 °C (one strain). In their work, only one strain out of 16 strains grew at temperatures below 8 °C. Rosso *et al.* [29] estimated 4.9 °C as the cardinal value of the minimum growth temperature for *E. coli.*

**Table 1:** Growth kinetics of primary models for a mixture of *E. coli* O157:H7^N^^R^ in blanched spinach and fresh-cut iceberg lettuce.

Temperature (°C)	LT ^x^					SGR ^y^				MPD ^z^			
	Spinach		Lettuce			Spinach		Lettuce		Spinach		Lettuce	
	Mean	SE	Mean		SE	Mean	SE	Mean	SE	Mean	SE	Mean	SE
7	63.36	1.67		52.8	0.80	0.004	0.00	0.004	0.04	7.20	0.02	7.14	0.06
10	28.32	0.26		40.08	0.06	0.005	0.00	0.005	0.03	7.31	0.25	7.23	0.20
17	10.80	0.87		8.88	0.09	0.006	0.00	0.008	0.00	7.81	0.03	8.04	0.02
24	3.60	0.13		4.28	0.21	0.019	0.01	0.015	0.05	8.06	0.04	8.18	0.08
30	2.64	0.05		2.64	0.34	0.049	0.00	0.051	0.00	8.04	0.03	8.27	0.13
36	2.40	0.19		2.40	0.02	0.110	0.02	0.092	0.02	8.80	0.14	8.41	0.17

**^x^** LT: Lag time (h); ^y^ SGR: Specific growth rate (log CFU/h); ^z^ MPD: Maximum population density (log).

In the present study, the growth of a mixture of *E. coli* O157:H7^NR^ in blanched spinach was observed at 7 °C after 63h of LT and was observed in TSBN after 105.6h of LT. On the contrary of nalidixic acid-resistant strains *E. coli* O157:H7, it is observed that the populations of *E. coli* O157:H7 decreased after 118 h of LT at 7 °C in this study (data not shown). The maximum population density (MPD) of a mixture of *E. coli* O157:H7^ NR^ in blanched spinach was reached at 7, 10, 17, 24, 30, and 36 °C on days of 12, 6, 3, 3, 1, and 1, respectively. However, the MPD of a mixture of *E. coli* O157:H7^ NR^ on blanched spinach and fresh-cut iceberg lettuce was significantly decreased at 7 and 10 °C (*p* < 0.05), indicating that the growth of nalidixic acid-resistant *E. coli* O157:H7 was inhibited during refrigerated storage. 

**Figure 2:**
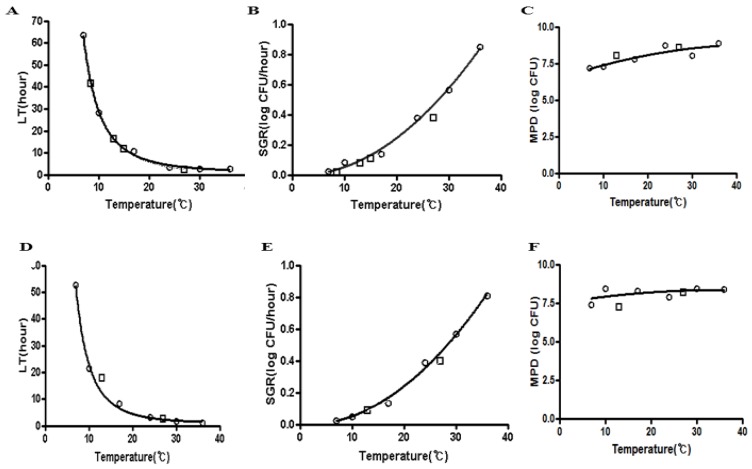
Secondary LT and SGR models of a mixture of *E. coli* O157:H7^NR^ in blanched spinach and fresh-cut iceberg lettuce as a function of temperature (7 to 36 °C). (**A**) Spinach-LT. (**B**) Spinach-SGR. (**C**) Spinach-MPD. (**D**) Lettuce-LT. (**E**) Lettuce-SGR. (**F**) Lettuce-MPD. ○ Dependent data □ Independent data.

Secondary models were developed to describe the primary model parameters, LT and SGR, as a function of temperature. The Davey model for LT, square root model for SGR, and polynomial model for MPD were used for blanched spinach and fresh-cut iceberg lettuce as a function of temperature, where growth was possible (Figure 2).

### 3.2. Evaluation of Model Performance of Secondary Spinach Models for Interpolation and Extrapolation (Model Extension)

The secondary models were first evaluated for the ability to interpolate within the response surface using independent temperature data not used in model development, which were collected using the same experimental design at 8.5, 13, 15 and 27 °C for blanched spinach and 13 and 27 °C for fresh-cut iceberg lettuce. Figure 2 shows that secondary models fit to the LT and SGR data of a mixture of *E. coli* O157:H7^NR^ in blanched spinach and fresh-cut iceberg lettuce as a function of temperature (7 to 36 °C). Both dependent and independent (interpolation) data were well fitted to the secondary models. Thus, the secondary models for LT and SGR had an acceptable goodness of fit and were validated for interpolation. Bias factor (*B_f_*) and accuracy factor (*A_f_*) with independent data were also calculated (Table 2). An acceptable range of *B_f_* of 0.7–1.15 and a range of 0.9–1.05 is considered to be good [16]. The LT, SGR, and MPD models for blanched spinach including interpolation data had *B_f_* of values of 1.01, 0.89, and 0.97, respectively, which indicated that the model predicted an LT, SGR, and MPD that was 1% lower, 11% higher, and 3% lower, respectively, than the actual experimental values.

**Table 2:** Performance of secondary growth models for a mixture of E. coli O157:H7^NT^ in blanched spinach and fresh-cut iceberg lettuce for interpolation.

Dataset	Model	B*_f_* ^a^	MRE ^b^	A*_f_* ^c^	MARE ^d^	%RE ^e^
Spinach	LT ^f^	1.01	0.01	1.16	0.14	90
	SGR ^g^	0.89	−0.13	1.15	0.12	100
	MPD ^h^	0.97	−0.02	1.04	0.03	100
Lettuce	LT ^i^	0.95	0.01	1.13	0.13	100
	SGR ^j^	0.96	−0.02	1.09	0.08	100
	MPD ^k^	1.00	0.00	1.02	0.02	100

^a^* B_f_*: bias factor; ^b^ MRE: median relative error; ^c^ *A_f_*: Accuracy factor; ^d^ MARE: mean absolute relative error; ^e^ %RE: percentage of relative error that is in an acceptable prediction zone from −30% to 15% for SGR, −60 to 0% for LT, and −80 to 40% for MPD; ^f^ LT = 2.00 + (−93.55/T) + (3,655.2/T^2^); ^g^ SGR = {0.0054(T − 1.177)}^2^; ^h^ MPD = 6.187 + 0.1377T − 0.001686T^2^; ^i^ LT = −0.12 + (−17.50/T) + (2,383.68/T^2^); ^j^ SGR = {0.0053(T − 1.263)}^2^; ^k^ MPD = 7.148 + 0.0350T + 0.0000633T^2^.

**Figure 3:**
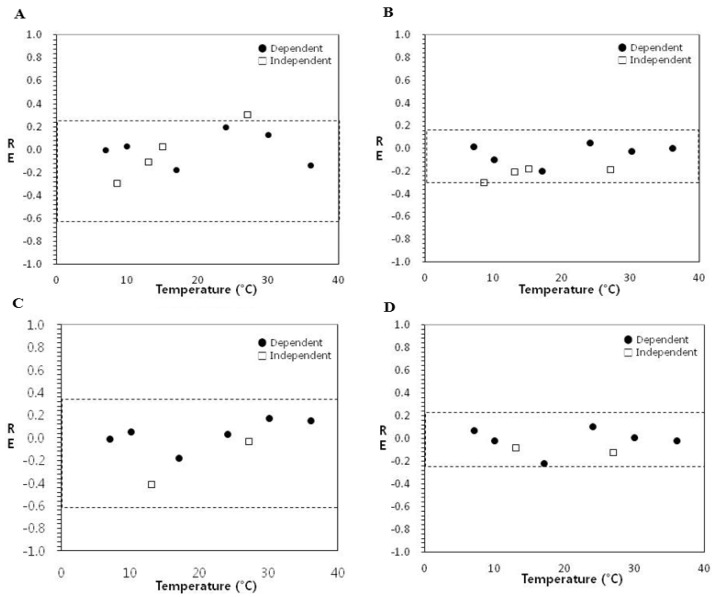
Acceptable prediction zone analysis of the goodness of fit of the secondary model for lag time (LT) and specific growth rate (SGR). RE values are mean (n=3). (**A**) Spinach-LT. (**B**) Spinach-SGR. (**C**) Lettuce-LT. (**D**) Lettuce-SGR.

Figure 3 shows the relative error (RE) plots with an acceptable prediction zone for lag time (LT) and specific growth rate (SGR). The LT model for an acceptable prediction zone was twice as wide (−0.6 to 0.3) as the acceptable prediction zone for the SGR model (−0.3 to 0.15) [15]. In the LT model of blanched spinach for dependent (model development) and independent (interpolation) data, 9 of 10 relative errors (percentage of RE = 90%) were inside the acceptable prediction zone (Figure 3(A)). On the other hand, all relative errors for SGR were inside the acceptable prediction zone (Figure 3(B)) and thus validated for interpolation (percentage of RE = 100%). Also, in the LT and SGR models for fresh-cut iceberg lettuce, all relative errors were inside the acceptable prediction zone (Figure 3(C,D)) and the performance was acceptable for interpolation (percentage of RE = 100%) as shown in Table 2.

**Figure 4:**
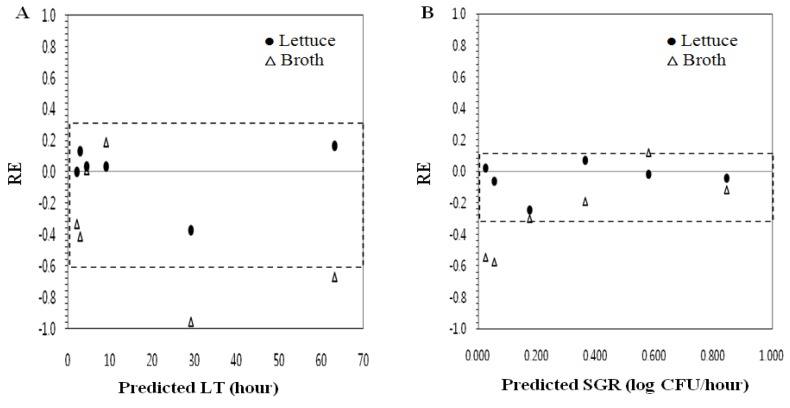
Relative error (RE) plots with an acceptable prediction zone analysis of lag time (LT) and specific growth rate (SGR) data used in evaluation of extrapolation. (**A**) LT. (**B**) SGR.

Model performance to test the possibility of extension of the model (extrapolation) to include other substrates was evaluated using independent sets of data obtained with different substrates. Fresh-cut iceberg lettuce and TSBN were used for extension for *E. coli* O157:H7^NR^ growth model for blanched spinach. In the LT model developed with blanched spinach, the *B_f_* and *A_f_* values for extrapolation to fresh-cut iceberg lettuce were 1.01 and 1.12, respectively (Table 3). The predictions of the LT model for extrapolation to fresh-cut iceberg lettuce were acceptable as six of six relative errors (RE) were inside the acceptable prediction zone (percentage of RE = 100%; Figure 4(A) and Table 3). On the other hand, the LT model for extrapolation to broth had a *B_f_* value of 0.77 and *A_f_* value of 1.40 (Table 3). These results indicated that the LT model developed for blanched spinach was unacceptable with broth (TSBN) model and suitable for fresh-cut iceberg lettuce. When the SGR model of blanched spinach was extended to iceberg lettuce, all relative errors (percentage of RE = 100%) were inside the acceptable prediction zone (Figure 4(B)) and had an acceptable *B_f_* value of 0.95 and *A_f_* value of 1.09. However, the SGR model for extension to TSBN had 50% relative errors (RE), and *B_f_* and *A_f_* values of 0.69 and 1.51, respectively (Table 3), indicating that the SGR model of blanched spinach was suitable to fresh-cut iceberg lettuce, but not suitable to TSBN, which was stored without agitation. Previous studies have reported that models prepared in liquid media may not give reliable predictions for growth of pathogens on foods [30,31,32]. Tamplin [30] observed immediate growth of *E. coli* in nonsterile ground beef, without the 49 h lag time predicted from the broth model of USDA Pathogen Modeling Program or the 12 h lag time predicted at 12 °C, reported by Walls and Scott [32]. Ross [31] also reported inhibition of MPD from *E. coli* in non-sterile minced beef, which was not observed in broth.

**Table 3:** Performance of secondary growth models for a mixture of E. coli O157:H7^NT^ in blanched spinach for extrapolation.

Dataset	Model	B*_f_* ^a^	MRE ^b^	A*_f_* ^c^	MARE ^d^	% RE ^e^
Lettuce	LT ^f^	1.01	0.03	1.12	0.12	100
	SGR ^g^	0.95	−3.04	1.09	0.08	100
Broth	LT	0.77	−0.37	1.40	0.43	33
	SGR	0.69	−24.68	1.51	0.31	50

^a^* B_f_*: Bias factor; ^b^ MRE: Median relative error; ^c^ *A_f_*: Accuracy factor; ^d^ MARE: Mean absolute relative error; ^e^ %RE: The percentage of relative error that is in an acceptable prediction zone from −30% to 15% for SGR and −60 to 30% for LT; ^f^ LT: Lag time (h); ^g^ SGR: Specific growth rate (log(CFU/h)).

**Figure 5:**
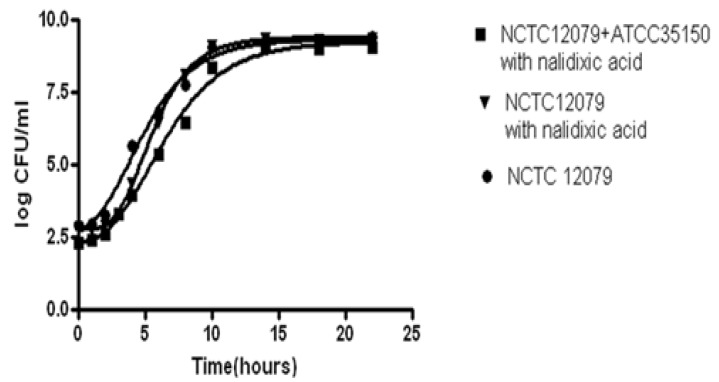
Comparison of growth curves of a mixture of two *E. coli* O157:H7^NR^ strains (NCTC12079^NR^ and ATCC35150^NR^) and *E. coli* O157:H7^NR^ strain (NCTC12079^NR^) and *E. coli* O157:H7 parent strain in broth at 36 °C.

A well-known strategy for modeling is to choose the fastest growing strain in the environmental conditions investigated, because the fastest growing strain will dominate the growth in food products [33]. McMeekin *et al.* [23] also recommended independent modeling of several different strains before choosing the strain that grows fastest under the environment conditions of most interest. Salter *et al.* [34] compared the growth of the nonpathogenic *E. coli* M23 with the growth of pathogenic strains of *E. coli* O157:H7 and found only small differences in the growth responses of these two strains. They also found that a model based on *E. coli* M23 was able to describe the growth of pathogenic strains of *E. coli* O157:H7. In the present study, we also compared the growth kinetics of a mixture of two *E. coli* O157:H7^NR^ strains (NCTC12079^NR^ and ATCC35150^NR^) to a single strain (NCTC12079^NR^) of *E. coli* O157:H7^NR^ and an *E. coli* O157:H7 parent strain (NCTC 12079). The growth kinetics of nalidixic acid-resistant strain of *E. coli* O157:H7 were not significantly different from those of *E. coli* O157:H7 parent strain in broth as the increase in temperature (Figure 5). 

## 4. Conclusions

The developed secondary LT, SGR, and MPD models were able to reliably predict the growth of a mixture of *E. coli* O157:H7^NR^ in blanched spinach, which were also suitable for use in making predictions for fresh-cut iceberg lettuce, but not for static broth (TSAN). In addition, the populations of *E. coli* O157:H7^NR^ increased in blanched spinach, fresh-cut iceberg lettuce, and broth at 7 °C, while the growth of parent *E. coli* O157:H7 strain was not observed in at 7 °C. These results indicate the risk of nalidixic acid-resistant strain of *E. coli* O157:H7 contamination in ready-to-eat produce at refrigerated temperatures. Additionally, before we used nalidixic acid-resistant strains of *E. coli* O157:H7 in this study to develop models for produces, their growth kinetics in broth at 7 to 36 °C were compared and found to be similar to those of parent *E. coli* O157:H7 strain from which they were derived. Thus the results of the current study also provide the growth characteristics of nalidixic acid-resistant *E. coli* O157:H7 in blanched and fresh-cut produces at various temperatures, which will be also useful information for microbial risk assessment and strategies for management of pathogenic *E. coli* in produce during distribution at the retail market level. 
